# Comprehension through cooperation: Medical students and physiotherapy apprentices learn in teams – Introducing interprofessional learning at the University Medical Centre Mannheim, Germany

**DOI:** 10.3205/zma001030

**Published:** 2016-04-29

**Authors:** Mira Mette, Mechthild Dölken, Jutta Hinrichs, Elisabeth Narciß, Katrin Schüttpelz-Brauns, Ute Weihrauch, Harald M. Fritz

**Affiliations:** 1 Medical Faculty Mannheim, Heidelberg University, Department of Undergraduate Education and Educational Development, Mannheim, Germany; 2 University Medical Centre Mannheim, School of Physiotherapy, Mannheim, Germany; 3 University Medical Centre Mannheim, Apprenticeship Training Centre, Mannheim, Germany

**Keywords:** Interprofessional education, interprofessional learning, medical education, physiotherapy, evaluation

## Abstract

**Aim:** In order to better prepare future health care professionals for interprofessional cooperation, interprofessional learning sessions for medical students and physiotherapy apprentices were developed at the University Medical Centre Mannheim, Germany. The experience gained from designing, implementing and evaluating these learning sessions is presented and discussed.

**Method: **A total of 265 medical students and 43 physiotherapy apprentices attended five interprofessional learning sessions. Of these, 87-100% responded to closed and open-ended questions on a self-developed questionnaire (24 items). The responses regarding self-reported learning gains, benefit, motivation and satisfaction with the sessions were analyzed separately by professions.

**Results: **The learning sessions were well received by both groups. More than 75% of all participants were of the opinion that they could not have learned the new material in a better way.

Significant differences between the medical students and the physiotherapy apprentices were mainly found with regard to perceived learning gains, which physiotherapy apprentices reported as being lower.

Positive aspects of interprofessionalism were most often emphasized in the responses to the open-ended questions. Most frequently criticized were organizational aspects and a lack of perceived learning gains.

**Conclusion: **The introduction of interprofessional learning entails great effort in terms of organizational and administrative challenges. However, the project is considered worthwhile because the interprofessional aspects of the learning sessions were indeed valued by the participants. Permanently including and expanding interprofessional learning in the curricula of both professions longitudinally is therefore something to strive for.

## Zusammenfassung

**Zielsetzung: **Um zukünftige Fachkräfte in der Patientenversorgung besser auf die interprofessionelle Zusammenarbeit vorzubereiten, wurden an der Universitätsmedizin Mannheim interprofessionelle Lerneinheiten für Medizinstudierende und Physiotherapieschüler entwickelt. Die Erfahrungen aus der Konzeption, Implementierung und Evaluation dieser Lerneinheiten werden dargestellt und diskutiert.

**Methoden:** An 5 interprofessionellen Lerneinheiten nahmen 265 Medizinstudierende und 43 Physiotherapieschüler teil. Von diesen beantworteten 87-100% im Rahmen der Lehrveranstaltungsevaluation geschlossene und offene Fragen eines selbstentwickelten Fragebogens (24 Items). Die Antworten zu selbstberichtetem Lernzuwachs, Nutzen, Motivation und Zufriedenheit mit der Lerneinheit wurden nach Profession getrennt ausgewertet.

**Ergebnisse: **Die Lerneinheiten wurden von beiden Teilnehmergruppen gut angenommen. Mehr als 75% aller Teilnehmer waren der Meinung, dass sie sich die neuen Lerninhalte nicht anderweitig besser hätten aneignen können.

Signifikante Unterschiede zwischen Medizinstudierenden und Physiotherapieschülern gab es vorrangig beim empfundenen Lernzuwachs, den die Physiotherapieschüler als geringer angaben.

In den offenen Fragen wurden am häufigsten interprofessionelle Aspekte positiv hervorgehoben. Kritisiert wurden am häufigsten organisatorische Punkte und der als gering empfundene Lernzuwachs.

**Schlussfolgerung:** Die Einführung von interprofessionellem Lernen ist durch organisatorische Herausforderungen sehr aufwändig. Es ist aber lohnenswert, da die interprofessionellen Aspekte der Lerneinheiten von den Teilnehmern als wertvoll angesehen wurden. Anzustreben ist deshalb eine curriculare Verstetigung und Erweiterung interprofessionellen Lernens zu einem longitudinalen Strang.

## Introduction

With a changing spectrum of diseases, ever more complex health care processes, and dwindling financial and human resources, the German health care system is faced with major challenges that demand closer cooperation between physicians and the other health care professionals to ensure the highest level of patient care and safety [http://www.bosch-stiftung.de/content/language1/html/44080.asp cited on 13 July 2015], [1]. Studies show that effective teamwork in health care can save lives, improve patient care, reduce errors, shorten hospital stays, and improve team communication and job satisfaction [2], [3]. The education of future physicians, nursing staff and therapists has up to now neglected to adequately prepare health care students and apprentices for interprofessional collaboration in later practice [http://www.bosch-stiftung.de/content/language1/html/44080.asp cited on 13 July 2015]. Acquiring interprofessional skills through interprofessional learning (IPL) is considered an effective means for enabling interprofessional collaboration in future practice [4]. The WHO views IPL as a necessary component of education in the health professions [5]. Even the German Council of Science and Humanities [1] recommends that all health care professionals seek to become qualified in terms of interprofessional collaboration, and this has been included in the recently approved German National Competency-Based Catalogue of Learning Objectives for Undergraduate Medical Education (NKLM) [6].

Due to widely differing conditions, the integration of IPL into curricula is very heterogeneous in the different countries. While for decades the United States, Canada, Australia, Great Britain, Scandinavia and Switzerland have assumed pioneering roles concerning IPL, in Germany initial attempts to address IPL are only now taking place at the post-secondary and university levels [7].

In the now considerable body of literature on IPL, it is primarily the development, piloting and evaluation of interventions using participant satisfaction as the measure of success that is described and discussed [8]. Studies able to underline the effectiveness of IPL in regard to improved patient outcomes are rare. In addition, the interventions described can hardly be transferred to other contexts due to the heterogeneity of the interprofessional course and seminar offerings [2], [7]. However, mixed groups of the smallest size possible and seminars focusing primarily on practice-relevant content have been demonstrated as being important for IPL [7].

This project report is not able to answer questions about the effectiveness of IPL, but using a concrete example it shows how IPL has been implemented for medical students and physiotherapy apprentices.

## Project description

Beginning in 2014 the Robert Bosch Stiftung provided a grant for a two-year interprofessional pilot project at the University Medical Centre Mannheim (UMM), Germany, involving cooperative learning in teams consisting of medical students and physiotherapy apprentices. The project, focusing on cooperative understanding and learning in teams, is a joint effort between the Medical Faculty Mannheim, Heidelberg University and the School of Physiotherapy at UMM.

During the pilot phase, an IPL approach was first proposed for medical students enrolled in the model study program MaReCuM and one other health profession. Physiotherapy was chosen not only because there are obvious points of intersection with medical studies and a broad range of possibilities for patient contact, but also for organizational aspects, such as the advantage of learning and doing internships on the same campus. Established, constructive relationships, not least with and among instructors who teach at both institutions, could be drawn upon while designing potential new IPL session offerings.

The aim of this pilot project is to connect the two educational programs using IPL at selected junctures. Joint learning sessions were developed and piloted for medical students (approx. 220 per year) and physiotherapy apprentices enrolled in the three-year physiotherapy program at the professional school (approx. 25 per year). The aim was to initiate dialogue between the two professional groups and impart interprofessional skills. Different, longitudinally integrated learning sessions ranging from anatomy to interactive exercises on interprofessional collaboration were designed with the goal of promoting the MaReCuM medical core competence of “working in a team for the benefit of patients,” based on the CanMEDS role of “Collaborator” [9] and the NKLM role of “the physician as a team member” [6].

The learning sessions focused on the three aspects of interprofessional learning defined by CAIPE [10]:

- Learning with each other: working on and expanding learning content together
- Learning from each other: imparting one’s own professional skills
- Learning about each other: acquiring knowledge about other professional groups

The interprofessional learning sessions are integrated longitudinally and systematically into the curricula of both educational programs over a number of years of study or apprenticeship so that all students and apprentices profit from it to the extent that organizational and legal constraints permit. This has been done to ensure that the topic is permanently embedded in both educational programs and the importance of the topic is highlighted.

### Interprofessional learning sessions

The learning sessions provide not only a chance to acquire interprofessional skills and engage in direct cognitive learning, but also to invite students to consider and even change their attitudes and behavior. As these sessions were being designed, teaching and learning methods that have proven to be successful in IPL studies were drawn upon, such as:

- Cooperative learning: working on assignments requires collaboration between both participant groups
- Experiential learning: newly acquired knowledge and skills are gained through interprofessional exchange
- Reflective learning: reflection in interprofessional groups allows for expression and understanding of the different occupational perspectives, as well as their competences and limits [11]

As the project’s focus indicates, an (inter)active practical element serves to connect the two professional groups in terms of content, while the joint reflection and discussion phase promotes interprofessional exchange. The instructors assume the role of facilitators who are there to clarify questions about content that cannot otherwise be answered in the interprofessional groups and to encourage targeted sharing and reflection.

Learning objectives were defined for each learning session based on the existing IPL competency frameworks, including those of the University of British Columbia [12], Canadian Interprofessional Health Collaborative [13], and the Interprofessional Education Collaborative (IPEC) Expert Panel [14]:

- Communication
- Teamwork
- Patient-centered health care
- Understanding one’s own role and responsibilities
- Knowledge of the specific roles and responsibilities of the other professions

**Figure 1:**
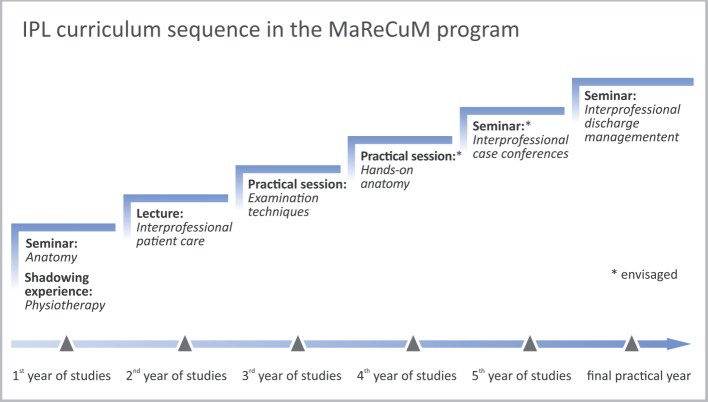
Illustration of the IPL curriculum sequence in the model study program MaReCuM

Figure 1 (Fig. 1) illustrates the sessions that were designed and developed (or are envisaged) as a coherent longitudinal IPL curriculum sequence in the model study program MaReCuM.

The learning sessions have been piloted since October 2014 and are required courses for both medical students and physiotherapy apprentices, with the exception of the anatomy seminar which is an elective for medical students.

#### Anatomy seminar

Medical students (1^st^ year) and physiotherapy apprentices (2^nd^ year) expand and deepen their knowledge of the musculoskeletal system using plastinates, skeletons and practical exercises.

#### Shadowing experience

As part of a placement in nursing in the hospital setting, medical students (1^st ^year) accompany physiotherapy apprentices (2^nd^ year and 3^rd ^year) as they work with patients to become familiar with the occupational field of physiotherapy.

#### Lecture on interprofessional patient care

Medical students (2^nd ^year) and physiotherapy apprentices (2^nd^ year) experience how professional groups work together in hospitals using a case example on which three instructors, each representing a different profession, interact.

#### Practical session on examination techniques

Medical students (3^rd^ year) and physiotherapy apprentices (3^rd^ year) demonstrate to each other and practice various techniques for examining the extremities and torso. 

#### Further interprofessional learning sessions

In September 2015 a seminar on **interprofessional discharge management** will take place in which IPL will be expanded to encompass nursing for the first time. Using actual cases, medical students (final practical year) and physiotherapy and nursing apprentices (both 2^nd ^year) will familiarize themselves with the interprofessional cooperation needed to discharge patients. Other practical sessions and seminars are envisaged.

#### Piloting and evaluation

The learning sessions combine the aspects of interprofessional learning [10] in different ways:

- Anatomy seminar: learning with, from and about each other
- Shadowing experience: learning from and about each other
- Lecture: learning about each other
- Practical session: learning with, from and about each other

Several organizational challenges had to be met during the development and implementation of the learning sessions. Above all, the different sizes of the two professional groups were problematic, since learning in small, heterogeneous groups is important for IPL [7]. To allow all of the medical students to observe the physiotherapy apprentices during the nursing placement, the shadowing experience had to take place on two days for the physiotherapy apprentices. Group rotations allowed the number of third-year medical students (220) to be broken down into groups of approximately 55 students who then learned together with around 20 physiotherapy apprentices. Also, the integration of the learning sessions into the existing course schedules of the seven-week module with mid-term and final exams was not easily accomplished, since IPL in the medical degree program was only able to occur as additional instruction. Due to legal regulations, the anatomy seminar in the first year of medical studies could not be offered as a mandatory course for all medical students and had to take place outside of the regular schedule of required courses.

Each learning session was evaluated upon completion. Since existing questionnaires, validated in terms of content and psychometrics, were not suitable (Readiness for Interprofessional Learning Scale, RIPLS) [15] or there was no validated German translation available (Interdisciplinary Education Perception Scale, IEPS), a self-developed, paper-based IPL standard questionnaire (24 items) was used between October 2014 and March 2015. This survey was anonymous with closed and open-ended questions on all four sections of the questionnaire:

- General questions
- Assessment of the learning session’s structure and organization
- Self-reported learning gains, benefit, motivation
- Overall evaluation and satisfaction with the learning session

The aim of the evaluation was quality assurance and improvement of the learning sessions, as well as identifying any differences between the two professional groups. The relevant items are presented in table 1 (Tab. 1).

A total of 265 medical students took part in the evaluation, of which five participated in two learning sessions, and 43 physiotherapy apprentices who, in part, participated repeatedly in different learning sessions. The composition of the participants per session is presented in table 2 (Tab. 2). The practical session was evaluated twice with regard to different topics (lower extremities vs. upper extremities). The evaluation data of the physiotherapy apprentices who participated more than once were not taken into account in the analysis.

Analysis of the data was carried out according to frequencies; the Mann-Whitney U test for independent samples was used for group comparisons.

## Results

The response rate depending on the learning session was 87-100%. The most important evaluation results are shown in table 3 (Tab. 3).

The mean rating for the learning sessions using the standard German grading scale (1=excellent, 6=very poor) was between 1.89 for the shadowing experience (both groups) and 2.49 for the practical session (medical students). The participants tended to agree that the importance of interprofessional collaboration became clearer to them through the learning sessions, that their interest in IPL had increased, and that they viewed the learning sessions as useful for their future professional work. In comparison to the other sessions, the agreement among the groups was lowest regarding the lecture. Despite this, the lecture was rated overall as “good”, similar to the other learning sessions. 

Significant differences between medical students and physiotherapy apprentices could only be determined in very few cases. For instance, as a result of the shadowing experience, the importance of interprofessional collaboration was clearer to the medical students than it was to the physiotherapy apprentices. In regard to the shadowing experience and the practical session, the self-reported learning gains for medical students was significantly higher than for the physiotherapy apprentices.

As table 4 (Tab. 4) illustrates, more than two-thirds of all participants in all of the learning sessions were of the opinion that they could not have learned the new content in a better way.

By asking open-ended questions, information indicating the level of participant satisfaction with the learning sessions was gathered, along with suggestions for improvement in future semesters. The open-ended responses to questions about what the participants particularly liked or disliked (multiple answers possible) were classified and quantified by topic. Above all, the participants valued the interprofessional aspects positively, with medical students emphasizing aspects related to subject-specific content more often, and physiotherapy apprentices mentioning social aspects of the joint sessions. Organizational details were criticized most often, but so was a lack of perceived learning gains (see table 5 (Tab. 5)).

## Discussion

The evaluation results show that the interprofessional learning sessions were generally well received by both professional groups. The goals set for the IPL sessions of becoming familiar with another professional group and gaining insights into other professions through sharing and understanding their professional perspectives were all positively highlighted. More than 75% of all participants were of the opinion that they could not have learned the new content in a better way. This indicates the approval of the new interprofessional sessions.

The learning sessions attempted to be as effective as possible by dividing students into small interprofessional groups with extensive focus on practical elements [7]. This was not possible in the lecture due to the teaching format and the disparate sizes of the two professional groups. Still, it was possible for three lecturers from different professions to clearly present an example of interprofessional collaboration in patient care.

The physiotherapy apprentices in particular criticized the lack of substantial learning gains in the shadowing experience and the practical session. This was to be expected for the shadowing experience since the focus was on learning from and about each other, and thus reciprocal learning was not intended. Less expected was the criticism voiced in regard to the practical session. Apparently, the physiotherapy apprentices understood learning gains to involve only subject-specific learning, while an increase in knowledge about other professions and how they work together with one’s own profession was not considered as learning. During the practical component it was revealed that for IPL the educational level of the participant groups must be taken into closer consideration, to enable joint learning on an equal level and to ensure that learning progress in the practical (subject-related) part is perceived by both groups [16]. Compared to the third-year medical students, it seems that the third-year physiotherapy apprentices were too advanced in terms of their educational level making input from both groups not as necessary when working together on the assignments.

Although the group rotation in the third year of the medical studies has advantages in respect to organization, it does have the disadvantage that the physiotherapy apprentices must take part more than once in specific learning sessions. The content of these sessions needs to be adjusted accordingly. A repetition of the shadowing experience is less problematic, since the physiotherapy apprentices regularly treat their patients with the only difference being that they are accompanied by a medical student and an interprofessional exchange takes place that, above all, assists medical students in reaching a better understanding of physiotherapy. To offer this kind of learning experience to physiotherapy apprentices, having them observe more advanced medical students, is currently being considered.

The information contained in the open-ended responses was used to optimize the learning sessions for later semesters. Changes include more interdependence of the groups in regard to assignments, stronger consideration of the educational levels of the groups, organizational improvements involving classroom space, meeting times, and practices for disseminating information. In general, it is necessary to determine at which points joint learning through interaction between medical students and physiotherapy apprentices makes so much sense that the organizational effort is worthwhile. For instance, the effort to coordinate course schedules and lecturers could be reduced by holding separate lectures for each professional group. In doing this, the interaction of the different professions in providing patient care could be presented by only one instructor. What speaks against doing this is that by using several instructors who each represent their own professions, the topic is presented in a much more authentic manner and most likely to have a more lasting effect as a result.

Overall, participant feedback confirmed that IPL using direct contact and personal exchange with another professional group brought new aspects to the educational program that the participants felt they could not otherwise have learned about in a better way. It must be noted though that the aim of the IPL curriculum sequence to better prepare all medical students and physiotherapy apprentices for interprofessional collaboration primarily depends on the support of the decision-makers and instructors at the medical faculty and their willingness to introduce the topic of IPL at specific points in medical education – preferably from third year onwards.

### Limitations

When considering the evaluations of the learning sessions, it must be taken into account that they involve self-reported data. In addition, there was no differentiation between subject-related and interprofessional learning gains. Moreover, the medical students attending the elective anatomy seminar were probably intrinsically motivated so that their evaluation data stems from a pre-selected and not a representative sample.

## Conclusion and Outlook

The implementation of IPL requires a significant amount of time and staffing for the organization, coordination, development and teaching of the learning sessions by instructors from different professions. The general conditions are not only specific to school location, but also inter- and intraprofessional in nature, making the incorporation of existing IPL concepts difficult. Still, the pilot project has been successful in introducing an IPL curriculum sequence of different learning sessions into the medical and physiotherapy programs at the UMM, with the goal of officially embedding them into both curricula and pursuing further development. New topics and other professional groups are to be successively integrated into IPL, for instance nursing apprentices joining medical students and physiotherapy apprentices in the seminar on discharge management. The placement of nursing for medical students at UMM is undergoing revision in collaboration with the nursing department and, starting in 2016, will include accompanying instruction covering interprofessional topics. In addition, other studies are planned to investigate the effectiveness of IPL sessions as well as the summative evaluation of the IPL curriculum sequence in the medical and physiotherapy programs.

## Funding

The project is funded by Robert Bosch Stiftung (project number 32.5.1316.0005.0).

## Acknowledgements

We wish to thank all of the IPL instructors for their commitment to developing and piloting the interprofessional sessions, along with the university administrators at the Medical Faculty Mannheim and UMM who gave their support and enabled us to offer IPL sessions. Our special gratitude goes to the Robert Bosch Stiftung, without whose support IPL at the UMM could not have happened.

## Competing interests

The authors declare that they have no competing interests.

## Figures and Tables

**Table 1:**
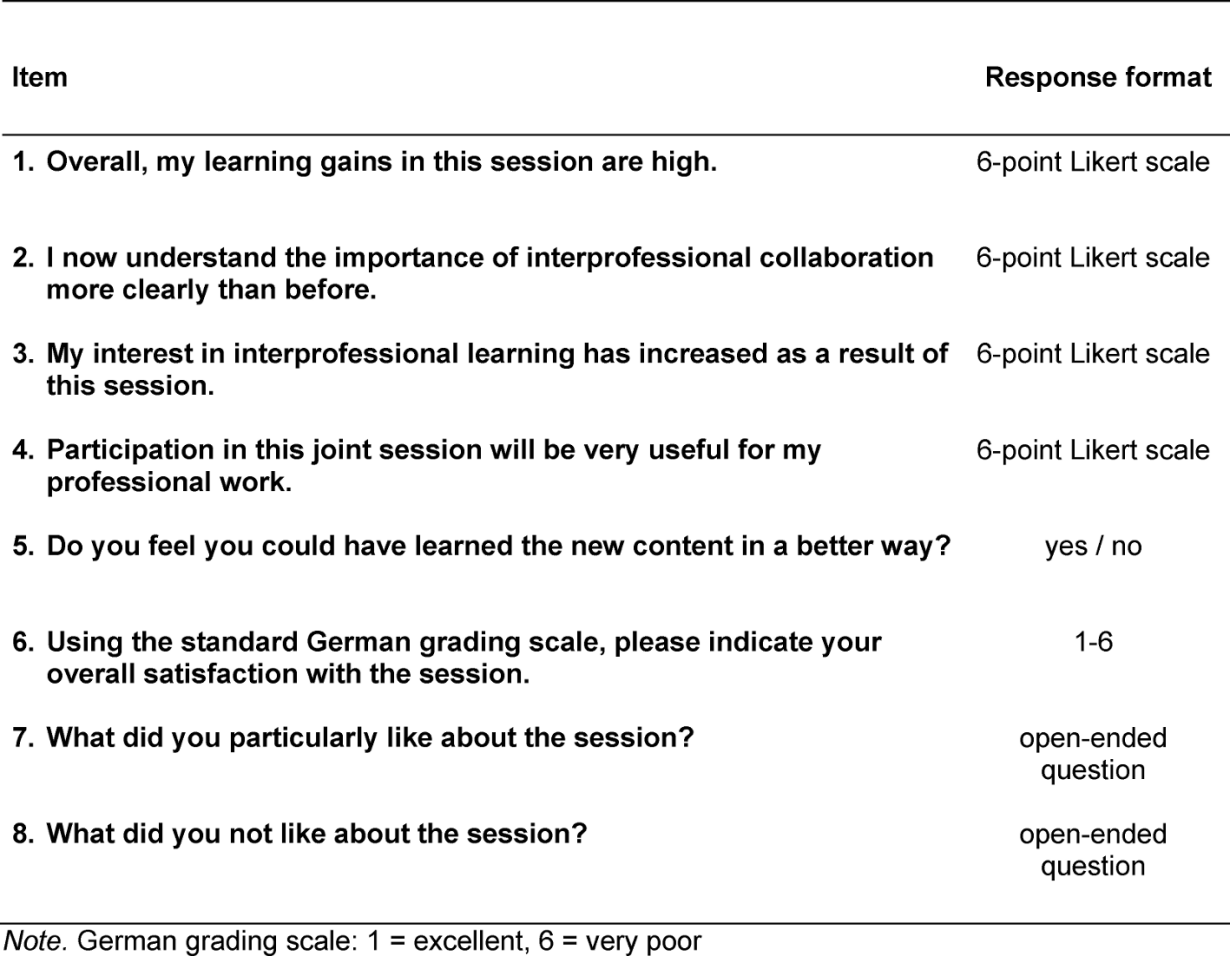
Excerpt from the IPL standard questionnaire: items on learning gains, benefit, motivation, overall evaluation and participant satisfaction

**Table 2:**
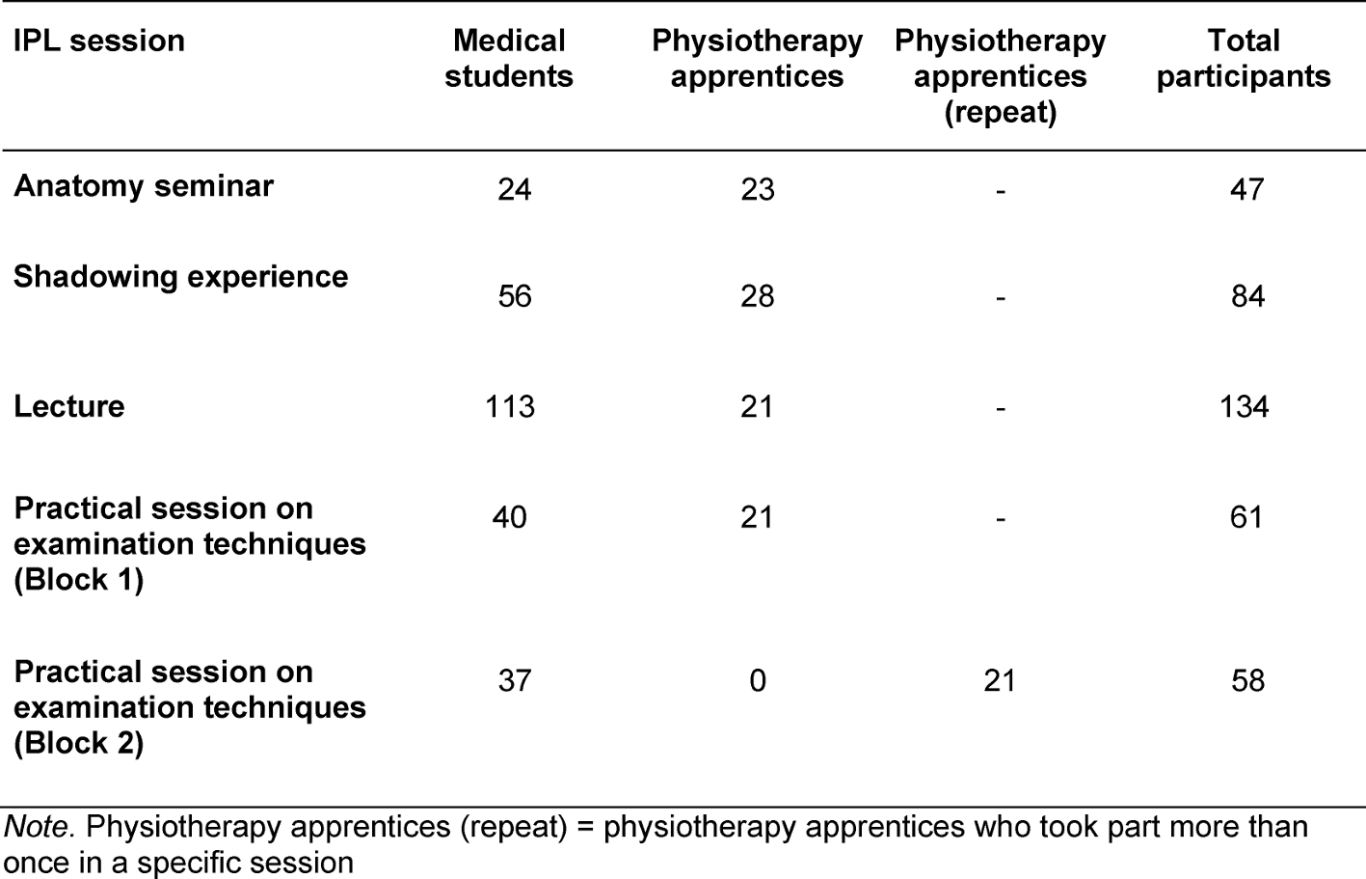
Composition of participants per learning session

**Table 3:**
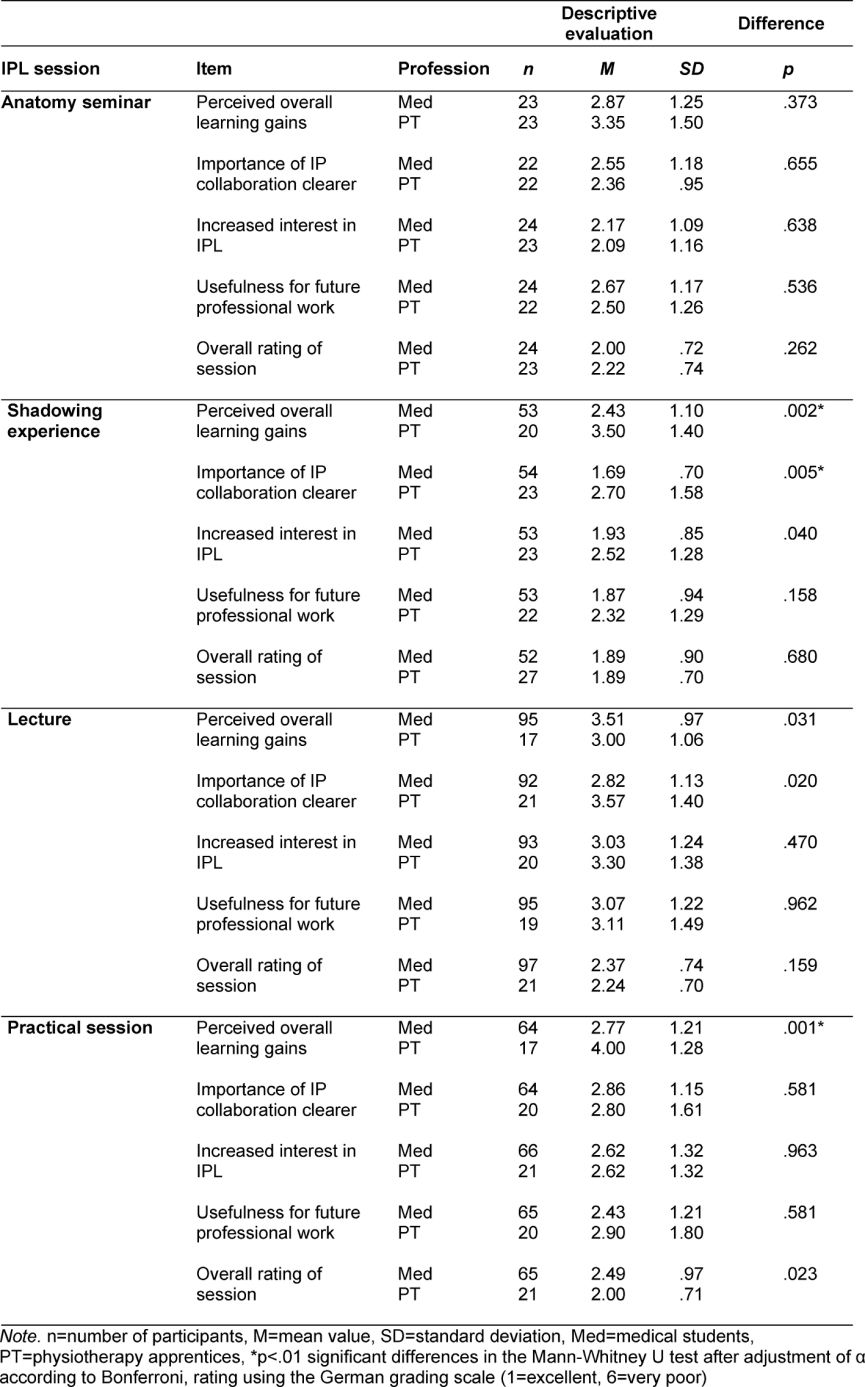
Evaluation results of the interprofessional learning sessions according to professional group

**Table 4:**
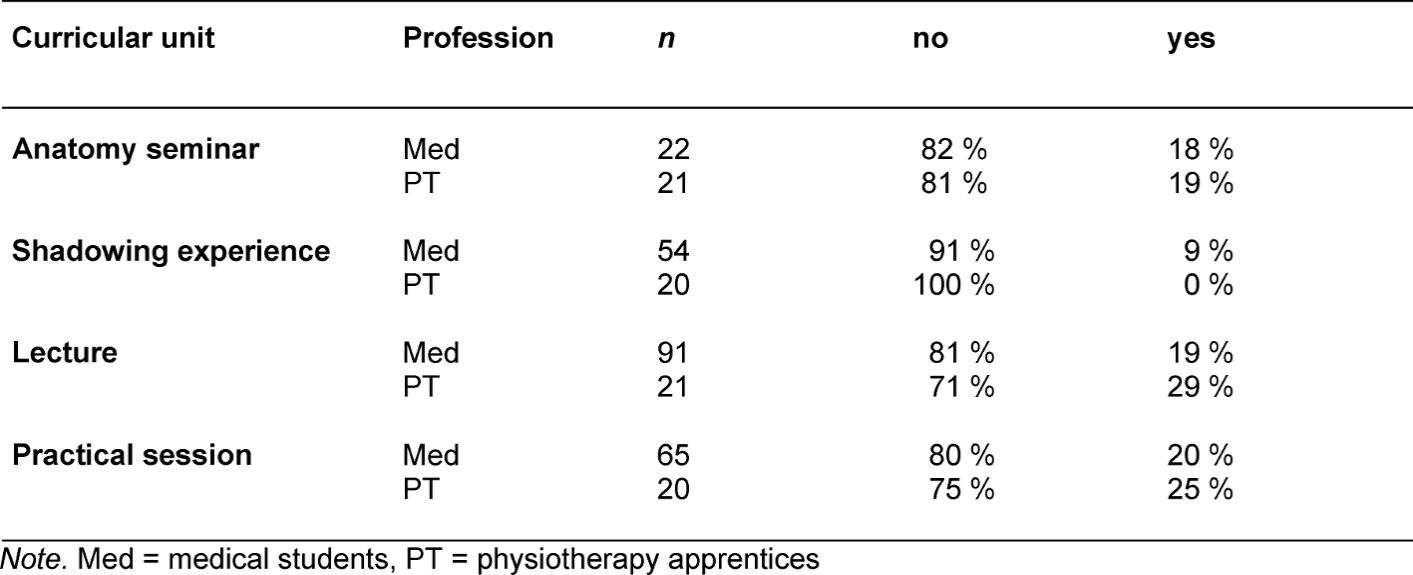
Evaluation results of the interprofessional learning sessions according to professional group for the item “Do you feel you could have learned the new content in a better way?”

**Table 5:**
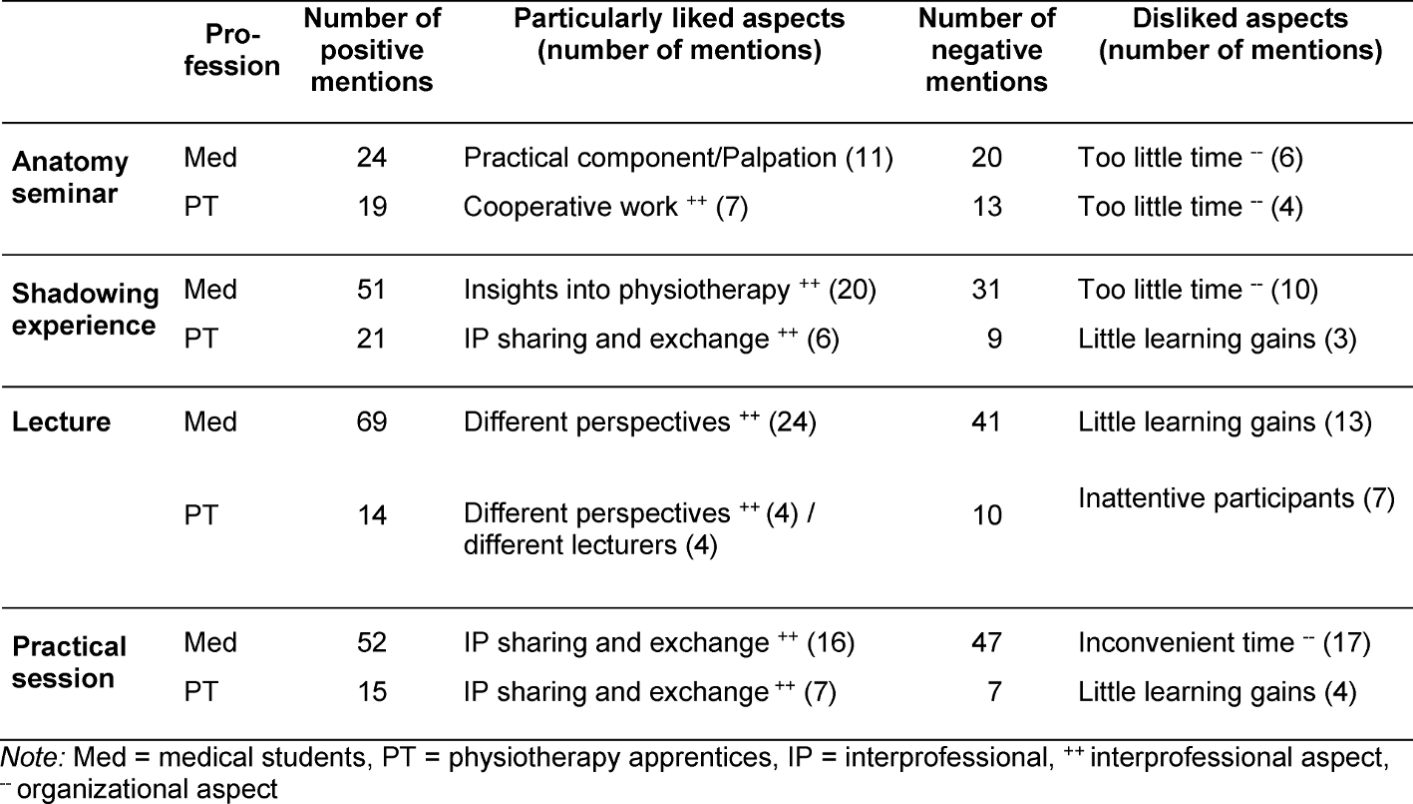
Presentation of the most frequent open-ended responses according to session and professional group

## References

[1] Wissenschaftsrat (2012). Empfehlungen zu hochschulischen Qualifikationen für das Gesundheitswesen.

[2] Reeves S, Zwarenstein M, Goldman J, Barr H, Freeth D, Koppel I, Hammick M (2010). The effectiveness of interprofessional education: Key findings from a new systematic review. J Interprof Care.

[3] Bharwani AM, Harris GC, Southwick FS (2012). Perspective: a business school view of medical interprofessional rounds: transforming rounding groups into rounding teams. Acad Med.

[4] Koch, LF (2012). Interprofessionelles Lehren und Lernen in den Ausbildungen der Gesundheits- und Sozialberufen. Eine Literaturanalyse zur Erarbeitung von Best-Practice-Empfehlungen für die Gestaltung interprofessioneller Bildungsangebote. http://dx.doi.org/10.3205/12gma007.

[5] World Health Organization (2010). Framework for Action on Interprofessional Education & Collaborative Practice.

[6] MFT Medizinischer Fakultätentag der Bundesrepublik Deutschland e (2015). V.

[7] Walkenhorst U, Mahler C, Aistleithner R, Hahn EG, Kaap-Fröhlich S, Karstens S, Reiber K, Stock-Schröer B, Sottas B (2015). Positionspapier GMA-Ausschuss - "Interprofessionelle Ausbildung in den Gesundheitsberufen". GMS Z Med Ausbild.

[8] Thistlethwaite J (2012). Interprofessional education: a review of context, learning and the research agenda. Med Educ.

[9] Frank JR (2005). The CanMEDS 2005 physician competency framework. Better standards. Better physicians. Better care.

[10] Centre for the Advancement of Interprofessional Education (2002). Interprofessional Education: A Definition.

[11] Clark PG (2006). What would a theory of interprofessional education look like? Some suggestions for developing a theoretical framework for teamwork training. J Interprof Care.

[12] College of Health Disciplines and Interprofessional Network of BC (2008). The British Columbia competency framework for interprofessional collaboration.

[13] Canadian Interprofessional Health Collaborative (2010). A National Interprofessional Competency Framework.

[14] IPECEP - Interprofessional Education Collaborative Expert Panel (2011). Core competencies for interprofessional collaborative practice: Report of an expert panel.

[15] Mahler C, Berger S, Reeves S (2015). The Readiness for Interprofessional Learning Scale (RIPLS): A problematic evaluative scale for the interprofessional field. J Interprof Care.

[16] Mette M, Dölken M, Magosch P, Hinrichs J, Narciß E, Schüttpelz-Brauns K, Fritz HM (2015). Evaluation des Lehrformats "Interprofessionelle Übungseinheit". http://dx.doi.org/10.3205/15gma193.

